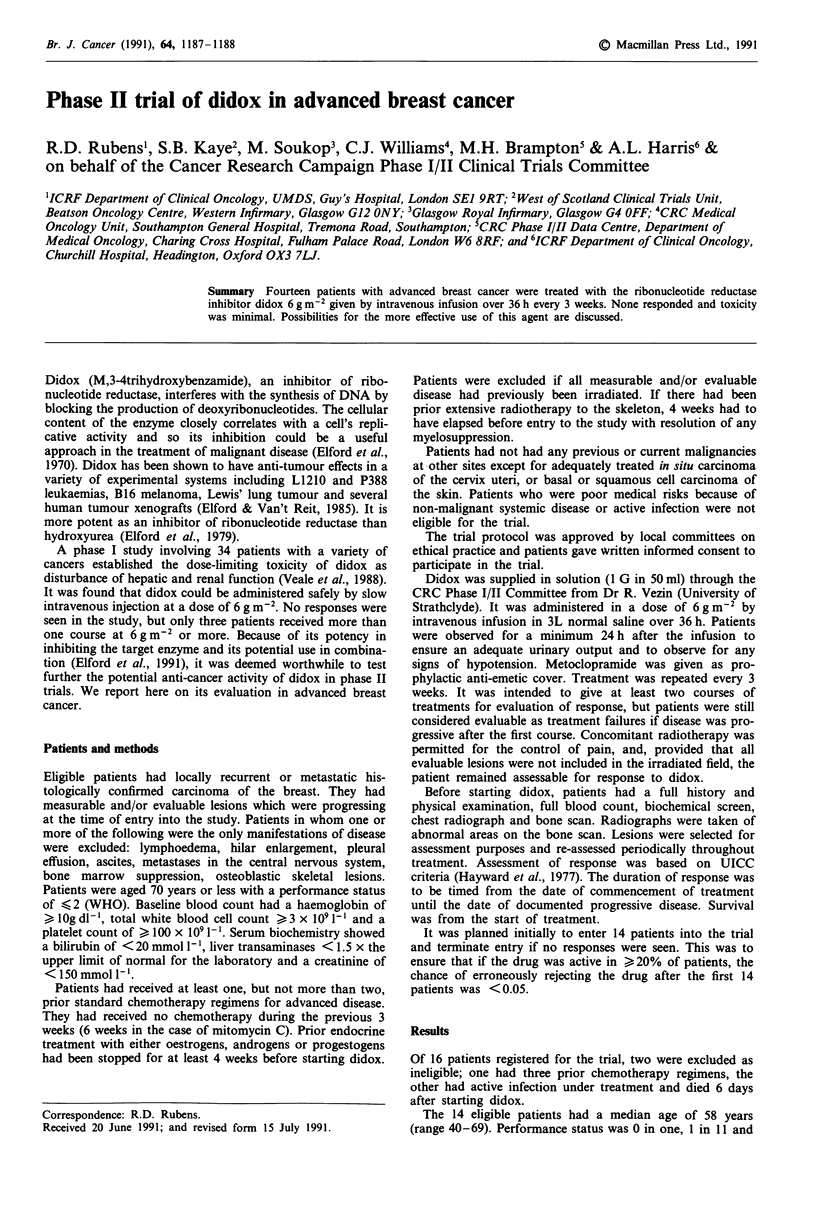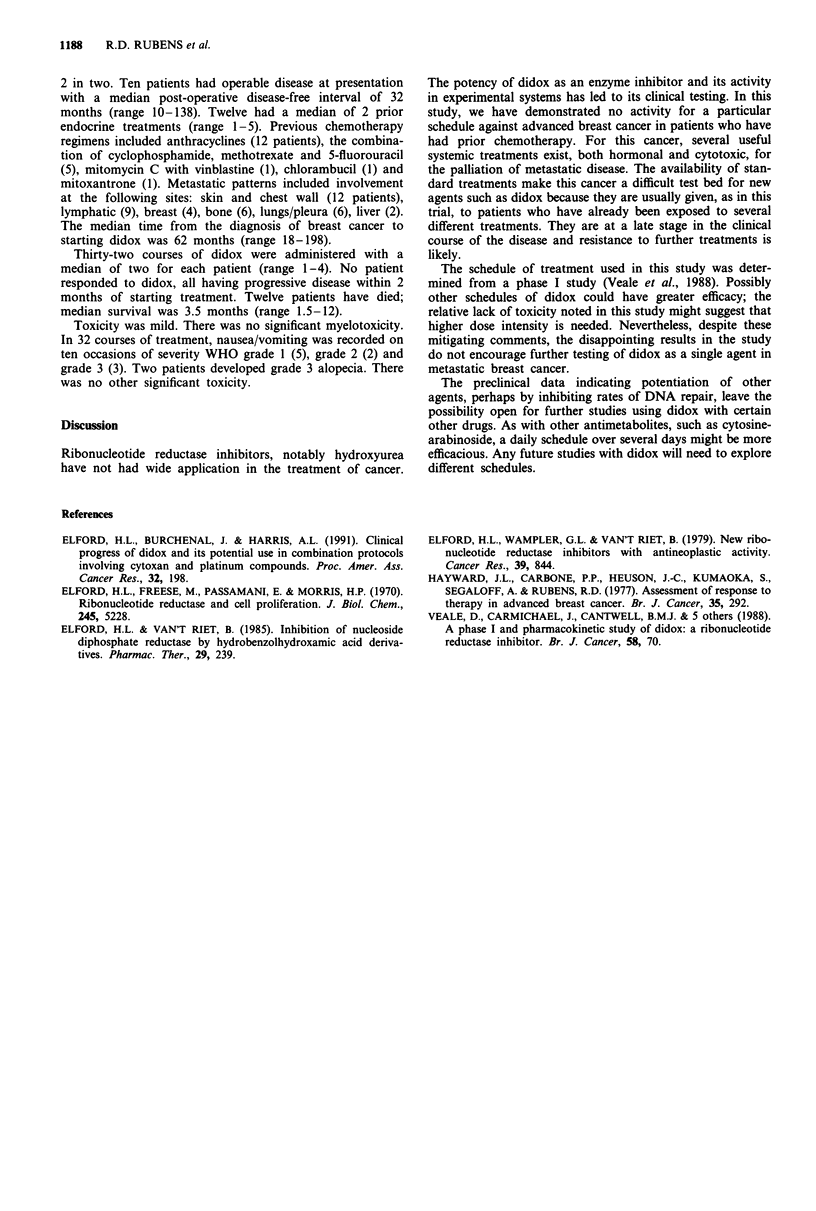# Phase II trial of didox in advanced breast cancer. Cancer Research Campaign Phase I/II Clinical Trials Committee.

**DOI:** 10.1038/bjc.1991.488

**Published:** 1991-12

**Authors:** R. D. Rubens, S. B. Kaye, M. Soukop, C. J. Williams, M. H. Brampton, A. L. Harris

**Affiliations:** ICRF Department of Clinical Oncology, UMDS, Guy's Hospital, London.

## Abstract

Fourteen patients with advanced breast cancer were treated with the ribonucleotide reductase inhibitor didox 6 g m-2 given by intravenous infusion over 36 h every 3 weeks. None responded and toxicity was minimal. Possibilities for the more effective use of this agent are discussed.


Br. J. Cancer (1991), 64, 1187-1188 © Macmillan Press Ltd., 1991

### Phase II trial of didox in advanced breast cancer

R.D. Rubens[1], S.B. Kaye[2], M. Soukop[3], C.J. Williams[4], M.H. Brampton[5] & A.L. Harris[6] & on behalf of the Cancer Research Campaign Phase I/II Clinical Trials Committee

[1]ICRF Department of Clinical Oncology, UMDS, Guy's Hospital, London SE1 9RT; [2]West of Scotland Clinical Trials Unit, Beatson Oncology Centre, Western Infirmary, Glasgow G12 0NY; [3]Glasgow Royal Infirmary, Glasgow G4 0FF; [4]CRC Medical Oncology Unit, Southampton General Hospital, Tremona Road, Southampton; [5]CRC Phase I/II Data Centre, Department of Medical Oncology, Charing Cross Hospital, Fulham Palace Road, London W6 8RF; and [6]ICRF Department of Clinical Oncology, Churchill Hospital, Headington, Oxford OX3 7LJ.

**Summary** Fourteen patients with advanced breast cancer were treated with the ribonucleotide reductase inhibitor didox 6 g m$^{-2}$ given by intravenous infusion over 36 h every 3 weeks. None responded and toxicity was minimal. Possibilities for the more effective use of this agent are discussed.

Didox (M,3-4trihydroxybenzamide), an inhibitor of ribonucleotide reductase, interferes with the synthesis of DNA by blocking the production of deoxyribonucleotides. The cellular content of the enzyme closely correlates with a cell's replicative activity and so its inhibition could be a useful approach in the treatment of malignant disease (Elford *et al.*, 1970). Didox has been shown to have anti-tumour effects in a variety of experimental systems including L1210 and P388 leukaemias, B16 melanoma, Lewis' lung tumour and several human tumour xenografts (Elford & Van't Reit, 1985). It is more potent as an inhibitor of ribonucleotide reductase than hydroxyurea (Elford *et al.*, 1979).

A phase I study involving 34 patients with a variety of cancers established the dose-limiting toxicity of didox as disturbance of hepatic and renal function (Veale *et al.*, 1988). It was found that didox could be administered safely by slow intravenous injection at a dose of 6 g m$^{-2}$. No responses were seen in the study, but only three patients received more than one course at 6 g m$^{-2}$ or more. Because of its potency in inhibiting the target enzyme and its potential use in combination (Elford *et al.*, 1991), it was deemed worthwhile to test further the potential anti-cancer activity of didox in phase II trials. We report here on its evaluation in advanced breast cancer.

### Patients and methods

Eligible patients had locally recurrent or metastatic histologically confirmed carcinoma of the breast. They had measurable and/or evaluable lesions which were progressing at the time of entry into the study. Patients in whom one or more of the following were the only manifestations of disease were excluded: lymphoedema, hilar enlargement, pleural effusion, ascites, metastases in the central nervous system, bone marrow suppression, osteoblastic skeletal lesions. Patients were aged 70 years or less with a performance status of $\leqslant 2$ (WHO). Baseline blood count had a haemoglobin of $\geqslant 10$g dl$^{-1}$, total white blood cell count $\geqslant 3 \times 10^9$ l$^{-1}$ and a platelet count of $\geqslant 100 \times 10^9$ l$^{-1}$. Serum biochemistry showed a bilirubin of $<20$ mmol l$^{-1}$, liver transaminases $<1.5 \times$ the upper limit of normal for the laboratory and a creatinine of $<150$ mmol l$^{-1}$.

Patients had received at least one, but not more than two, prior standard chemotherapy regimens for advanced disease. They had received no chemotherapy during the previous 3 weeks (6 weeks in the case of mitomycin C). Prior endocrine treatment with either oestrogens, androgens or progestogens had been stopped for at least 4 weeks before starting didox.

Patients were excluded if all measurable and/or evaluable disease had previously been irradiated. If there had been prior extensive radiotherapy to the skeleton, 4 weeks had to have elapsed before entry to the study with resolution of any myelosuppression.

Patients had not had any previous or current malignancies at other sites except for adequately treated *in situ* carcinoma of the cervix uteri, or basal or squamous cell carcinoma of the skin. Patients who were poor medical risks because of non-malignant systemic disease or active infection were not eligible for the trial.

The trial protocol was approved by local committees on ethical practice and patients gave written informed consent to participate in the trial.

Didox was supplied in solution (1 G in 50 ml) through the CRC Phase I/II Committee from Dr R. Vezin (University of Strathclyde). It was administered in a dose of 6 g m$^{-2}$ by intravenous infusion in 3L normal saline over 36 h. Patients were observed for a minimum 24 h after the infusion to ensure an adequate urinary output and to observe for any signs of hypotension. Metoclopramide was given as prophylactic anti-emetic cover. Treatment was repeated every 3 weeks. It was intended to give at least two courses of treatments for evaluation of response, but patients were still considered evaluable as treatment failures if disease was progressive after the first course. Concomitant radiotherapy was permitted for the control of pain, and, provided that all evaluable lesions were not included in the irradiated field, the patient remained assessable for response to didox.

Before starting didox, patients had a full history and physical examination, full blood count, biochemical screen, chest radiograph and bone scan. Radiographs were taken of abnormal areas on the bone scan. Lesions were selected for assessment purposes and re-assessed periodically throughout treatment. Assessment of response was based on UICC criteria (Hayward *et al.*, 1977). The duration of response was to be timed from the date of commencement of treatment until the date of documented progressive disease. Survival was from the start of treatment.

It was planned initially to enter 14 patients into the trial and terminate entry if no responses were seen. This was to ensure that if the drug was active in $\geqslant 20\%$ of patients, the chance of erroneously rejecting the drug after the first 14 patients was $<0.05$.

### Results

Of 16 patients registered for the trial, two were excluded as ineligible; one had three prior chemotherapy regimens, the other had active infection under treatment and died 6 days after starting didox.

The 14 eligible patients had a median age of 58 years (range 40-69). Performance status was 0 in one, 1 in 11 and

Correspondence: R.D. Rubens.
Received 20 June 1991; and revised form 15 July 1991.

2 in two. Ten patients had operable disease at presentation with a median post-operative disease-free interval of 32 months (range 10–138). Twelve had a median of 2 prior endocrine treatments (range 1–5). Previous chemotherapy regimens included anthracyclines (12 patients), the combination of cyclophosphamide, methotrexate and 5-fluorouracil (5), mitomycin C with vinblastine (1), chlorambucil (1) and mitoxantrone (1). Metastatic patterns included involvement at the following sites: skin and chest wall (12 patients), lymphatic (9), breast (4), bone (6), lungs/pleura (6), liver (2). The median time from the diagnosis of breast cancer to starting didox was 62 months (range 18–198).

Thirty-two courses of didox were administered with a median of two for each patient (range 1–4). No patient responded to didox, all having progressive disease within 2 months of starting treatment. Twelve patients have died; median survival was 3.5 months (range 1.5–12).

Toxicity was mild. There was no significant myelotoxicity. In 32 courses of treatment, nausea/vomiting was recorded on ten occasions of severity WHO grade 1 (5), grade 2 (2) and grade 3 (3). Two patients developed grade 3 alopecia. There was no other significant toxicity.

### Discussion

Ribonucleotide reductase inhibitors, notably hydroxyurea have not had wide application in the treatment of cancer. The potency of didox as an enzyme inhibitor and its activity in experimental systems has led to its clinical testing. In this study, we have demonstrated no activity for a particular schedule against advanced breast cancer in patients who have had prior chemotherapy. For this cancer, several useful systemic treatments exist, both hormonal and cytotoxic, for the palliation of metastatic disease. The availability of standard treatments make this cancer a difficult test bed for new agents such as didox because they are usually given, as in this trial, to patients who have already been exposed to several different treatments. They are at a late stage in the clinical course of the disease and resistance to further treatments is likely.

The schedule of treatment used in this study was determined from a phase I study (Veale *et al.*, 1988). Possibly other schedules of didox could have greater efficacy; the relative lack of toxicity noted in this study might suggest that higher dose intensity is needed. Nevertheless, despite these mitigating comments, the disappointing results in the study do not encourage further testing of didox as a single agent in metastatic breast cancer.

The preclinical data indicating potentiation of other agents, perhaps by inhibiting rates of DNA repair, leave the possibility open for further studies using didox with certain other drugs. As with other antimetabolites, such as cytosine-arabinoside, a daily schedule over several days might be more efficacious. Any future studies with didox will need to explore different schedules.

### References

ELFORD, H.L., BURCHENAL, J. & HARRIS, A.L. (1991). Clinical progress of didox and its potential use in combination protocols involving cytoxan and platinum compounds. *Proc. Amer. Ass. Cancer Res.*, **32,** 198.

ELFORD, H.L., FREESE, M., PASSAMANI, E. & MORRIS, H.P. (1970). Ribonucleotide reductase and cell proliferation. *J. Biol. Chem.*, **245,** 5228.

ELFORD, H.L. & VAN'T RIET, B. (1985). Inhibition of nucleoside diphosphate reductase by hydrobenzolhydroxamic acid derivatives. *Pharmac. Ther.*, **29,** 239.

ELFORD, H.L., WAMPLER, G.L. & VAN'T RIET, B. (1979). New ribonucleotide reductase inhibitors with antineoplastic activity. *Cancer Res.*, **39,** 844.

HAYWARD, J.L., CARBONE, P.P., HEUSON, J.-C., KUMAOKA, S., SEGALOFF, A. & RUBENS, R.D. (1977). Assessment of response to therapy in advanced breast cancer. *Br. J. Cancer*, **35,** 292.

VEALE, D., CARMICHAEL, J., CANTWELL, B.M.J. & 5 others (1988). A phase I and pharmacokinetic study of didox: a ribonucleotide reductase inhibitor. *Br. J. Cancer*, **58,** 70.